# Baseline incidence of adverse birth outcomes and infant influenza and pertussis hospitalisations prior to the introduction of influenza and pertussis vaccination in pregnancy: a data linkage study of 78 382 mother–infant pairs, Northern Territory, Australia, 1994–2015

**DOI:** 10.1017/S0950268819001171

**Published:** 2019-07-04

**Authors:** L. McHugh, R.M. Andrews, B. Leckning, T. Snelling, M.J. Binks

**Affiliations:** 1 Menzies School of Health Research, Charles Darwin University, Tiwi, Northern Territory, Australia; 2 National Centre for Epidemiology & Population Health, Australian National University, Canberra, Australian Capital Territory, Australia; 3 Curtin University, School of Public Health, Perth, Western Australia; 4 University of Western Australia Perth, Western Australia; 5 Wesfarmers Centre of Vaccines and Infectious Diseases, Telethon Kids Institute, Perth, Western Australia, Australia; 6 Perth Children's Hospital, Perth, Western Australia

**Keywords:** Aboriginal and Torres Strait Islander, Australia, hospitalisations, incidence, infants, influenza, pertussis

## Abstract

We conducted probabilistic data linkage of three population datasets for the Northern Territory (NT), Australia, to describe the incidence of preterm births, stillbirths, low birthweight and small for gestational age (SGA) per 1000 NT births; and influenza and pertussis hospitalisations per 1 00 000 NT births in infants <7 months of age, in a pre-maternal vaccination era. The Perinatal Trends dataset (1994–2014) formed the cohort of 78 382 births. Aboriginal mother–infant pairs (37%) had disproportionately higher average annual rates (AR) for all adverse birth outcomes compared to their non-Aboriginal counterparts; rate ratios: preterm births 2.2 (AR 142.4 *vs.* 64.7); stillbirths 2.3 (AR 10.8 *vs.* 4.6); low birthweight 2.9 (AR 54 *vs.* 19); and SGA 1.7 (AR 187 *vs.* 111). Hospitalisation (2000–2015) and Immunisation Register datasets (1994–2015), showed that influenza hospitalisations (*n* = 53) and rates were 42.3 times higher in Aboriginal infants (AR 254 *vs.* 6); and that pertussis hospitalisations (*n* = 37) were 7.1 times higher in Aboriginal infants (AR 142.5 *vs.* 20.2) compared to non-Aboriginal infants. These baseline data are essential to assess the safety and effectiveness of influenza and pertussis vaccinations in pregnant women from the NT. Remote living Aboriginal women and infants stand to benefit the most from these vaccines.

## Introduction

Globally, pregnant women, particularly indigenous pregnant women and their infants, experience severe health consequences from influenza and pertussis infections compared to the general population [1, 2]. Two vaccines are routinely recommended in pregnancy in Australia to combat the high disease burden from these infections; inactivated influenza vaccine (IIV) and diphtheria-Tetanus-acellular pertussis (dTpa) [3]. Seasonal IIV was first recommended in pregnancy for Australian women in 2000, although not funded (provided for free) until 2010, [3] whilst dTpa (pertussis) vaccination in pregnancy was recommended and funded by the states and territories in 2015 [4].

Despite these recommendations, systematic programs to monitor the uptake, safety and effectiveness of IIV and pertussis vaccines in pregnancy remain absent. To determine the safety and effectiveness of vaccination in pregnancy, it is critical to quantify the baseline rates of adverse birth outcomes and the disease burden from influenza and pertussis infections in early infancy in a *pre*-maternal vaccination uptake era. A World Health Organization (WHO) report has stressed that baseline information on the safety of immunisation during pregnancy is essential in order to distinguish between vaccine benefits and safety concerns in local populations [5].

In Australia, pregnant women and Aboriginal and/or Torres Strait Islander (hereafter respectfully referred to as Aboriginal*) women and their infants are over-represented in notification and hospitalisation rates and intensive care unit (ICU) admissions for influenza and pertussis infections [6, 7]. The Northern Territory (NT) of Australia is geographically vast (1.42 million km^2^) yet has a relatively small population (~245 000) who are substantively younger and compared to the rest of Australia, have the highest population proportion who identify as Aboriginal [8, 9]. Small studies have found Aboriginal women and infants living in remote and very remote regions of the NT incur disproportionately higher rates of adverse birth outcomes compared to non-Aboriginal women and infants, [10] and little is known about the severity and geographic distribution of influenza and pertussis infections in infants spanning the introduction of IIV and pertussis vaccination in pregnancy.

Note: *In the Northern Territory, ‘Aboriginal’ is the preferred official term for inclusively referring to both Aboriginal and Torres Strait Islander peoples. This respectfully acknowledges the relatively small percentage of the NT population who identify as Torres Strait Islanders and the greater proportion of these people who also identify as Aboriginal [11].

In the NT between 2008 and 2012, laboratory-confirmed influenza infection notification rates were consistently highest in women of child-bearing age and Aboriginal children in the 0–<5 years age group [12]. Despite the higher disease burden of influenza in these groups and recommendations, uptake of IIV in pregnancy amongst NT women was very low (~<5%) prior to the 2009 pandemic [13] and has remained low thereafter at ~30% from 2014 [14]. Influenza vaccination is not licensed, nor recommended for use in infants <6 months of age. These infants can only be protected against influenza infection via maternal antibody transfer if IIV is given to the mother during pregnancy [15]. Several randomised controlled trials and observational studies have demonstrated that IIV in pregnancy is effective in reducing influenza infection in pregnant women and infants [16–18].

For infants to be considered protected against pertussis infection, the Australian National Immunisation Program (NIP) currently recommends a three-dose primary infant pertussis vaccination schedule; at 6–8 weeks, at 4 months and at 6 months of age [3]. Despite this schedule\ and high levels of vaccine uptake (>90%) in infancy, [19] pertussis notifications and hospitalisations remain highest in infants <4 months of age, [20] and are consistently higher in Aboriginal infants compared to non-Aboriginal infants [21, 22]. As a result of the high mortality and morbidity in infants too young to be fully protected from routine immunisation, administration of a pertussis-containing vaccine was recommended in the UK and the USA for all women in the third trimester of every pregnancy to confer early infant protection, via maternal antibody transfer [23, 24]. This strategy was found to be ~95% effective in reducing pertussis infections in infants <4 months of age in these countries [25, 26]. Based on these findings, the maternal pertussis vaccination program was recommended in Australia and subsequently implemented in the NT from April 2015 [4].

We aimed to describe the baseline incidence of adverse birth outcomes for the whole of the NT population by year of infant birth, remoteness and Indigenous status; we also aimed to describe the baseline incidence, severity and geographic variation of influenza and pertussis hospitalisations in infants from birth to 6 months of life by Indigenous status and region in a *pre*-maternal vaccination uptake era.

## Methods

### Study design

We used three de-identified, unit-record linked whole of NT population administrative datasets from the Developmental Outcomes of Children in the NT study, [11] to conduct retrospective cohort analyses.

### Study population

Our cohort encompassed all recorded NT births from 01 January 1994 to 31 December 2014 inclusive. This time period meant the birth cohort was expected to be vaccine naïve for either IIV or pertussis vaccines in pregnancy. The maternal pertussis vaccination program did not commence until April 2015 and there was a very small proportion of IIV uptake in pregnancy recorded during the 2009 H1N1 influenza pandemic until 2011 (<5%) [13]. Uptake of IIV in pregnancy in the NT is lacking for 2012 and there was an estimated uptake of ~15–30% from 2013 onwards [14, 27].

### Data sources

1. The NT Perinatal Trends dataset (perinatal dataset) encompassed a whole of NT population birth cohort from 01 January 1994 to 31 December 2014 inclusive. These data were used to establish the study cohort. Missing data were treated as such.
2. The NT Hospital Inpatient Activity dataset (hospitalisation dataset) records all episodes of care for patients admitted to all NT hospitals. Our dataset captured all episodes of hospitalisations from 01 July 2000 to 31 December 2015 inclusive. We examined infant hospitalisations where a diagnosis of influenza and/or pertussis was recorded, based on the Australian modified International Statistical Classification of Diseases and Related Problems (ICD-10-AM) codes, for infants born in the NT whose mother had a record in the perinatal dataset [28].
3. The NT Immunisation register (NTIR) captured all episodes of vaccinations administered in the NT from 01 January 1994 to 31 December 2015. Immunisation status was ascertained for recommended scheduled vaccines in hospitalised infants with an ICD-10-AM diagnosis of influenza and pertussis.

### Data linkage and preparation

A mix of deterministic and probabilistic linkage was used to match records from NT administrative datasets in health, education and welfare services for all children born between 01 January 1994 and 31 December 2014. This established the whole cohort for the Developmental Outcomes of Children in the NT study [11] and became the data repository from which we drew our study population. A unique project-specific linkage key (PSLK) was generated for each child in the repository, which enabled linkage of individual records across all datasets in the repository. The perinatal dataset defined our study cohort and the PSLK was used to link these infants to their hospitalisation and immunisation records as needed for analysis. Duplicate records for infants in the hospitalisation and NTIR datasets were identified. Mothers in the perinatal dataset had a unique identifier which could be used to distinguish multiple and subsequent births, including siblings. Both the NT and Commonwealth Departments of Health (DoH) report Indigenous status of infants based on the Indigeneity of the mother. As such, we have assigned Indigeneity of the infant the same way [29, 30].

#### Remoteness

Australian Bureau of Statistics (ABS) geographical classifications were used to compare demographic characteristics and birth outcomes by degrees of remoteness in the NT: Outer Regional, Remote and Very remote [31]. The NT has no major cities or inner regional areas by ABS definition. Comparisons of infant influenza and pertussis hospitalisations were conducted between the two major health service regions of the NT: Top End and Central Australia (CA) due to the large geographical distance between the two (~1400 km) and distinctly different climates. The Top End is considered monsoonal with two seasons-dry and wet, whilst CA has extreme temperature variations and a dry, arid environment.

#### Inclusion/exclusion criteria

Infants in the hospitalisation and immunisation datasets who had no perinatal record were excluded from the cohort. Infants were excluded from being ‘at-risk’ of a hospitalisation if they had no record in the immunisation dataset indicating at least one vaccination episode in the first 12 months of life. Our reasoning was that evidence of health service activity in the 12 month period after birth constituted ongoing NT residency. Hospitalisation and immunisation records were excluded from the study cohort if infants were >12 months of age at the time of the encounter.

### Statistical methods and data analysis

The perinatal dataset was used to calculate proportions for maternal and infant demographic characteristics, maternal comorbidities and risk factors and birth outcomes and to calculate the incidence of adverse birth outcomes over the 20-year observation period. These factors were analysed by Indigenous status, region and remoteness. For continuous variables, means and medians were calculated depending on the distribution of the data.

#### Birth outcomes

The birth outcomes of interest were: preterm births, stillbirths, low birthweight infants at term (LBWT) and small for gestational age (SGA). Infants born before 37 completed weeks' gestation were defined as preterm, [30] and stillborn infants were defined as showing no signs of life after a pregnancy of at least 20 weeks gestation or weighing 400 g or more [32]. Infant birthweight <2500 grams was defined as low birthweight (LBW) in accordance with the WHO definition [33]. As preterm birth is the main contributor to LBW [34], we restricted LBW to include singleton infants born at term; 37 completed weeks' gestation (LBWT). Small for gestational age (birthweight lower than the 10th percentile), was calculated using Australian national birthweight percentiles by sex and gestational age [35].

#### Infant hospitalisations

Following data linkage, the infant outcomes of interest were the proportion and rate of hospitalisation for influenza and pertussis infections, calculated by year of infant birth, Indigenous status and remoteness for the first 6 completed months of life. Of specific interest was the number and rate of infant hospitalisations for influenza during the 2009 influenza A (H1N1) pandemic. The following ICD-10-AM codes were used when searching for diagnoses in all hospitalisation episodes: influenza, J09-J11; pertussis, A37- A37.9. Infant age in weeks at hospital admission, number of days hospitalised, ICU admissions and geographic distribution of hospitalisation were calculated.

#### Average annual incidence rates

Average annual incidence rates (AR) for preterm births, stillbirths, LBWT and SGA were calculated per 1000 NT births, by year of infant birth, Indigenous status and remoteness. Rate ratios were calculated to compare rates between Aboriginal and non-Aboriginal (referent group) infants. Average annual incidence rates (AR) of infant influenza and pertussis hospitalisations were calculated per 1 00 000 NT population for infant age in weeks at time of hospitalisation, by year of infant birth, Indigenous status and by region. Rate ratios were calculated to compare rates between Aboriginal and non-Aboriginal (referent group) infants. As hospitalisations were only captured from 01 July 2000 onwards, infants born before 2000 were excluded from these analyses to prevent truncation bias. The total number of infant births from 01 July 2000 to 30 July 2014 was used as the denominator to calculate infant hospitalisation rates in weeks. Data were analysed using Stata v.14.1 (StataCorp, College Station, Texas) and graphs and figures were constructed using Microsoft Excel and Stata v.14.1.

#### Ethics approvals

This project was approved by the NT DoH/ Menzies School of Health Research (Menzies) Human Research Ethics Committee (HREC) reference number: 2017–2749, the DoH/Menzies Top End HREC, reference number: 2015–2333, the Central Australian HREC, reference number: 15–293, the Steering Committee of the Developmental Outcomes of Children in the NT study comprising key NT government agencies and the Aboriginal Medical Services Alliance of the NT (AMSANT). A stipulation of ethics approvals precluded the reporting of cell sizes <5.

## Results

### Study cohort

**Fig. 1.**
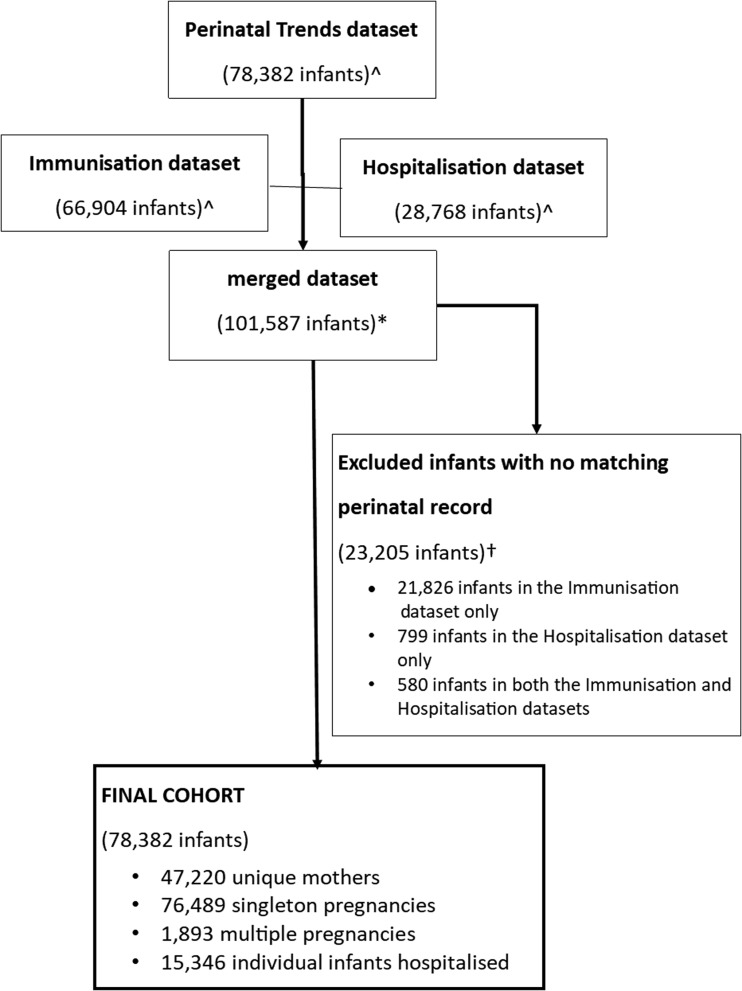
Data linkage study cohort for Northern Territory mothers and infants, Australia 1994–2015, inclusive. ^Individual infants included in the respective datasets who were born 1994–2015 inclusive. *Merged dataset contained uniquely identified infants from one or more of the available datasets, linked using Project Specific Linkage Key. †Infants were excluded from the final cohort if they did not have a linked perinatal record.

Based on the perinatal dataset, 47 220 individual women birthed 78 382 infants between 01 April 1994 and 31 December 2014 (Fig. 1). Data on maternal Indigenous status was >99% complete and 28 715 (37%) of infants had mothers who identified as an Aboriginal woman.

Most births were singleton (*n* = 76 489, 98%) with no difference between Aboriginal and non-Aboriginal mothers (98% *vs.* 97%). There were 1 878 sets of twins (2%) and 15 sets of triplets (<1%). We excluded 2 665 infants who did not have a record showing at least one vaccination episode in the first 12 months of life as per protocol because of lack of evidence of ongoing NT residency. There were 56 903 infant hospitalisations; 13 350 newborn admissions and 10 852 admissions for infants aged 7 months or older were not included as per protocol. Of the remaining 33 141 hospitalisations, Indigenous status was known for 32 701 infants (99%) and of these infants, 23 042 (70%) were Aboriginal.

### Demographic characteristics

Most mother–infant pairs (63%) were from Outer regional areas; 37% were from Remote/Very remote areas. The mean maternal age of women at infant birth was 27 years (range 12–51 years) and 53% presented for antenatal care in the first trimester of pregnancy. Compared to Outer regional living women, women who lived in Remote/Very remote areas were younger, less likely to present for antenatal care in the first trimester and more likely to experience co-morbidities and have high-risk factors. Most women (74%), were Aboriginal compared to 16% non-Aboriginal (Tables S1 and S2).

### Birth outcomes

**Fig. 2.**
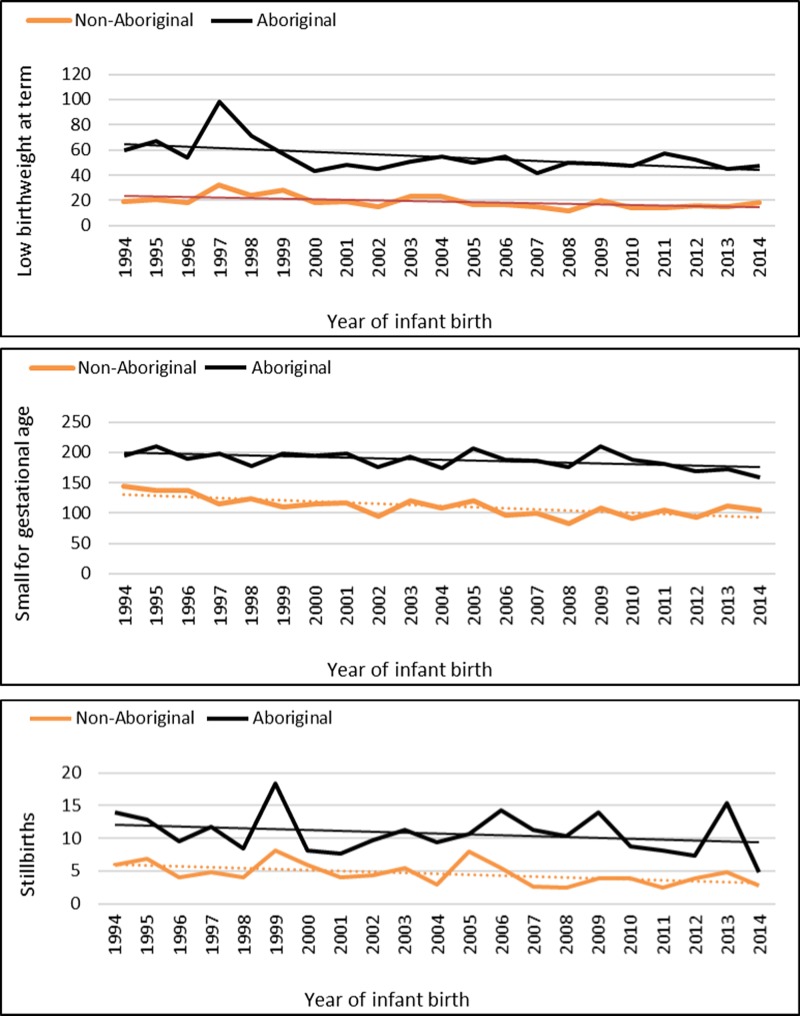
Low birthweight, small for gestational age and stillbirth rates per 1000 Northern Territory births, Australia (1994–2014).

**Fig. 3.**
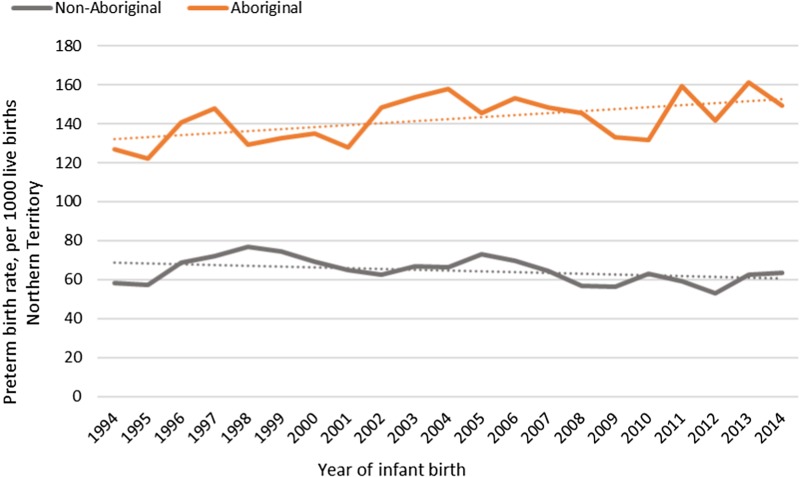
Preterm birth rates per 1000 Northern Territory births, Australia (1994–2014).

The median gestational age at birth of infants was 39 weeks and mean birthweight was 3253 grams. There was no clinically important variation in these birth outcomes by Indigenous status (Table 1) however, for preterm births, LBWT, SGA and stillbirths, proportions and AR were consistently higher for Aboriginal women compared to non-Aboriginal women (Table 1). In both groups, there was a decreasing trendline throughout the study period for LBWT, SGA and stillbirths (Fig. 2), however, the decreasing trend for preterm births that was observed for non-Aboriginal women was incongruent to an increasing trend for Aboriginal women (Fig. 3).

**Table 1.** Birth outcomes and average annual rates^a^ for Northern Territory women and infants, by Indigenous status, Australia (1994–2014)

Characteristics *N* (%) Total 76 489	Aboriginal (*N* = 28 158)	Non-Aboriginal (*N* = 48 309)	Incident rate ratios^f^ (range)
Median gestation in weeks (range)	39 (20–44)	39 (20–44)	n/a
Mean birthweight in grams (range)	3098 (400–5834)	3355 (400–5980)	n/a
Remote living	20 258 (74%)	7717 (16%)	n/a
Preterm birth^b^	4014 (14%)	3117 (6%)	
Average annual rate	142.4	64.7	2.2 (1.7–2.7)
Low birthweight at term^c^	1352 (5%)	986 (2%)	
Average annual rate	54	19	2.9 (2.0–4.2)
Small for gestational age^d^	5240 (19%)	5165 (11%)	
Average annual rate	187	111	1.7 (1.3–2.1)
Stillbirths^e^	399 (1.39%)	321 (0.65%)	
Average annual rate	10.8	4.6	2.3 (1.3–4.3)

Note: Results do not include data for 22 infants with unknown Indigenous status. Singleton births only.

a Rates per 1000 Northern Territory births.

b <37 completed weeks gestation.

c <2500 grams and ⩾37 weeks gestation.

d <10th percentile for Australian infants by sex.

e ⩾20 weeks gestation and ⩾400grams.

f Referent group- non-Aboriginal infants.

### Influenza and pertussis hospitalisations

Among 51 297 infants born in the 2000–2015 cohort, 15 346 (30%) were hospitalised at least once (33 141 episodes) before the age of 7 months. There were 57 infants hospitalised for influenza (0.2%) and 41 for pertussis (0.1%). There were nine deaths and 67% of these were Aboriginal infants from Remote/Very remote regions of the NT (data not shown elsewhere).

#### Influenza

**Fig. 4.**
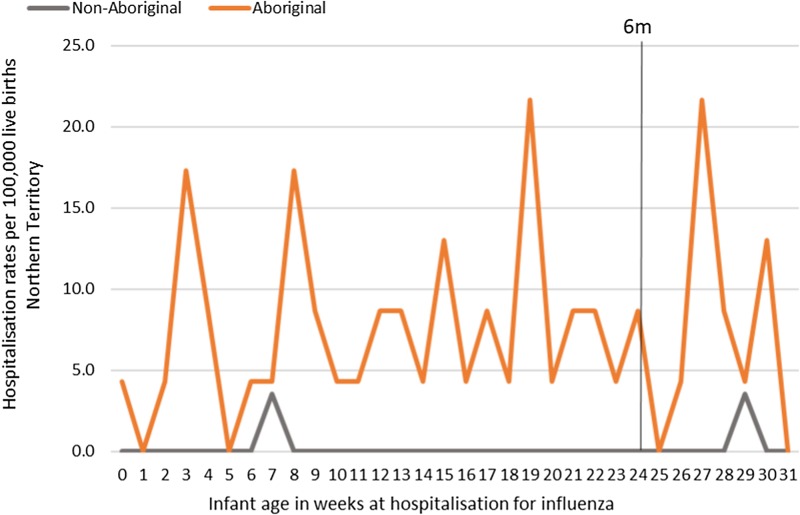
Influenza hospitalisation rates per 100 000 live births for infants 0–<7 months old, by week of infant birth, Northern Territory, Australia (2000–2014).

Most infants hospitalised for influenza were Aboriginal (*n* = 51, 96%), with a median age at admission of 17 weeks (range <1–30) and 91% (*n* = 48) lived in a Remote/Very remote setting. More infants were hospitalised with influenza in the Top End (*n* = 36, 68%) than in Central Australia (CA) (*n* = 17, 32%) and the majority of these infants were Aboriginal (34/36, 94%), (Table 2). The AR of influenza hospitalisations was 43 (range 0–46.4) times higher in Aboriginal infants compared to non-Aboriginal infants (259 *vs.* 6), (Fig. 4).

**Table 2.** Influenza hospitalisations for Northern Territory infants 0–<7 months of life, by region (2000–2015)

Characteristics	Total *N* = 53	Top End *n* = 36/53 (68%)	Central Australia *n* = 17/53 (32%)
Aboriginal	51 (96%)	34 (67%)	17 (33%)
Age at admission in weeks^a^^,^^b^ (range)	17 (<1–30)	14 (2–30)	22 (<1–30)
Length of stay in days^a^ (range)	5 (1–339)	5 (1–339)	5 (1–55)
ICU admissions	<5 cells^c^	0	<5 cells^c^
Remote/very remote living	48 (91%)	32/48 (67%)	16/48 (33%)
Preterm	11 (21%)	9 (82%)	<5 cells^c^
Low birthweight at term	<5 cells^c^	0	<5 cells^c^
SGA	<5 cells^c^	<5 cells^c^	<5 cells^c^

a Median values, based on non-parametric distribution of data.

b Censored at 6 completed months of infant life.

c Actual numbers not shown if cells had <5 results.

#### 2009 Influenza A (H1N1) pandemic

**Fig. 5.**
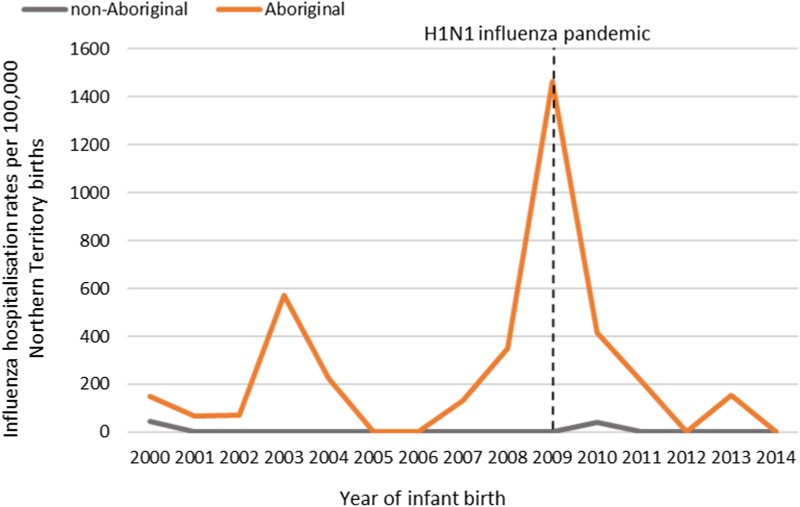
Influenza hospitalisation rates in 0–<7 month old infants, by year of infant birth in the Northern Territory, Australia (2000–2014).

The NT recorded its first case of H1N1 pandemic influenza in May 2009 [36]. Subsequently, 2009 saw the highest number of infant influenza hospitalisations recorded throughout the study period (22/57, 39%; Figure 5). All 22 of these hospitalisations occurred in Aboriginal infants, with 19/22 (86%) among infants living in Remote areas and 13/22 (59%) born during the Southern Hemisphere influenza season.

#### Pertussis hospitalisations

**Fig. 6.**
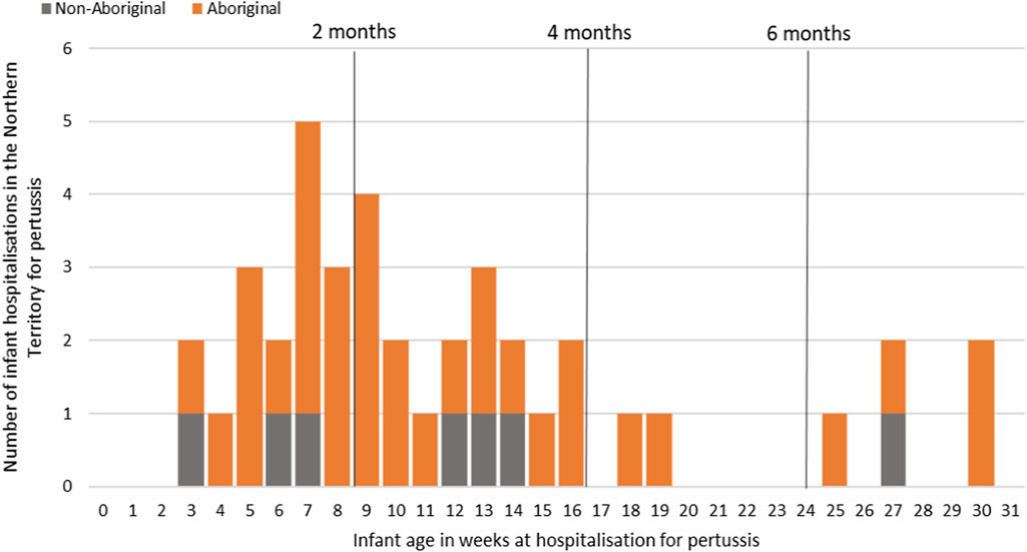
Number of pertussis hospitalisations in 0–<7 month old infants, by age in weeks of infant at time of hospitalisation and recommended pertussis vaccination schedule, Northern Territory, Australia (2000–2014).

**Fig. 7.**
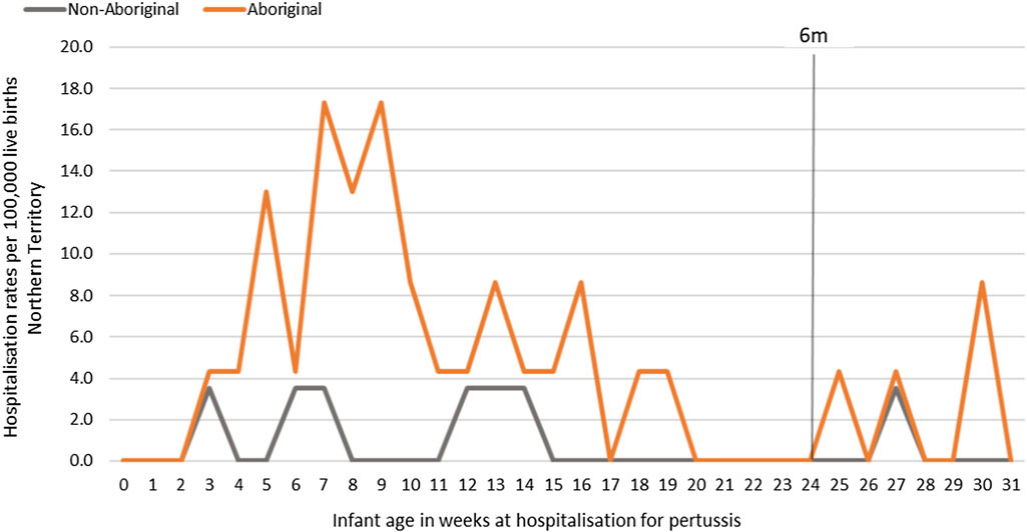
Pertussis hospitalisation rates per 100 000 live births in 0–<7 month old infants, by year of infant birth at time of hospitalisation, Northern Territory, Australia (2000–2014).

**Fig. 8.**
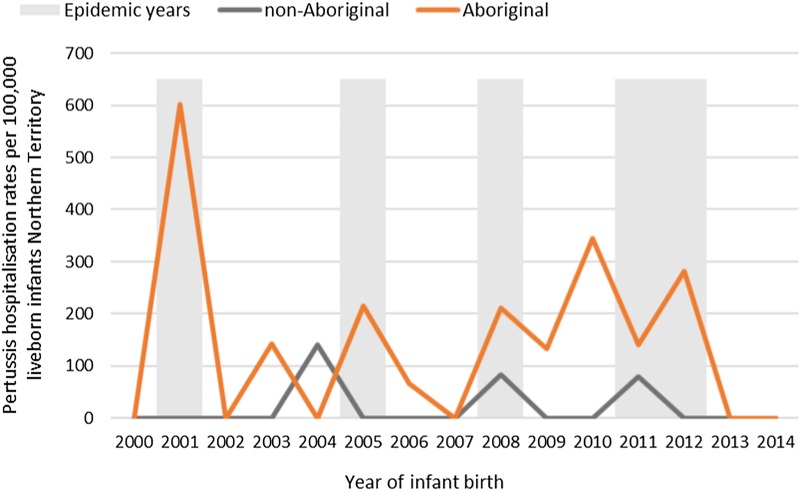
Pertussis hospitalisation rates per 100 000 live births in 0–<7 month old infants, by age in weeks of infant at time of hospitalisation, Northern Territory, Australia (2000–2014).

Of the 41 pertussis hospitalisations, four infants (10%) had unknown Indigenous status and none had received a pertussis-containing vaccine as part of their infant schedule prior to hospitalisation. Of the remaining 37 infants with a known Indigenous status, most hospitalised for pertussis were Aboriginal (*n* = 30/37, 81%); had not received any pertussis-containing vaccines prior to hospitalisation (30/37, 81%), were Remote/Very remote living (*n* = 25/37, 68%) (Table 3). Nearly all Aboriginal infants had an older sibling, (26/30, 87%) compared to less than half of non-Aboriginal infants (3/7, 43%). Most pertussis hospitalisations occurred predominantly in the first 4 months of life (Fig. 6). The AR of pertussis hospitalisations was 7.1 (range 0–17.1) times higher among Aboriginal infants compared to non-Aboriginal infants (142.5 *vs.* 20.2), particularly evident during the national epidemic years 2001, 2008 and 2011 (Figs 7 and 8).

**Table 3.** Pertussis hospitalisations for Northern Territory infants 0–<7 months of life, by Indigenous status and region (2000–2015)

Characteristics	Total *N* = 37	Top End *n* = 24/37 (65%)	Central Australia *n* = 13/37 (35%)
Aboriginal	30 (81%)	19 (63%)	11 (37%)
Age at admission in weeks^a^^,^^b^ (range)	11 (3–30)	11 (3–30)	10 (6–16)
Length of stay in days^a^ (range)	5 (1–48)	5 (1–48)	4 (1–19)
ICU admissions	<5 cells^c^	<5 cells^c^	0
Pertussis vaccination status^d^	7 (19%)	7/24 (29%)	0
Remote/very remote living	25 (68%)	19/25 (76%)	6/25 (24%)
Preterm	6 (16%)	6/37	0
Low birthweight at term	<5 cells^c^	<5 cells^c^	<5 cells^c^
SGA	12 (21%)	6/12 (50%)	6/12 (50%)

Note: excludes four infants with unknown Indigenous status and unknown region.

a Median values, based on non-parametric distribution of data.

b Censored at 6 completed months of infant life.

c Actual numbers not shown if cells have <5.

d Percent vaccinated with a pertussis-containing vaccine prior to hospitalisation.

## Discussion

This is the largest linked whole of population cohort of 78 382 mother–infant pairs from the NT of Australia to date, of which a substantial proportion (~37%) are Aboriginal. There are few whole of population studies with comparable long term data on Aboriginal and remote-living populations. Baseline observed incidence rates of adverse birth outcomes and infant influenza and pertussis hospitalisations in a *pre-*maternal vaccination period were all disproportionately higher amongst Aboriginal infants than non-Aboriginal infants. These data are critical in order to monitor disease surveillance, assess vaccine effectiveness and evaluate the safety of the NT maternal influenza and pertussis vaccination programs. Given the size of our cohort, these data will provide a valuable future reference.

### Birth outcomes

Though there was a disparity in the incidence for all adverse birth outcomes between Aboriginal and non-Aboriginal women and infants, apart from preterm births, there was a reassuring downward trend over the observation period. The upward trend in preterm births experienced by Aboriginal women is concerning. It is unclear whether this upward trend is an artefact of improvements in survival rates of very preterm and extremely preterm infants who historically experienced higher mortality rates or a true increase due to persisting high levels of co-morbidities and risk factors among Aboriginal women. Remoteness of living is a known risk factor for adverse birth outcomes including preterm birth [10], and our data supports this. Women with the highest preterm birth rates were Remote living Aboriginal women, who also had more co-morbidities and risk factors recorded for preterm birth, such as low attendance of first-trimester antenatal care, maternal anaemia, urinary tract infections, sexually transmissible infections and smoking. Socioeconomic factors play a major role in these high rates of co-morbidities and risk factors for Aboriginal women and an improvement in the quality and appropriateness of antenatal care provided to Remote living Aboriginal women has been recommended as a way of reducing these disproportionately high adverse birth outcomes [37].

### Infant influenza and pertussis hospitalisations

Our results confirm the heavy burden of illness from influenza infection in young Aboriginal infants, particularly during pandemic years. There is potential to reduce the number of infant hospitalisations, the length of stay in hospital and ICU admissions, particularly among Remote living Aboriginal infants under 6 months of age, by improving the uptake of IIV in pregnancy [38, 39]. The uptake of IIV in pregnancy remains poor in the NT and Australia wide, [40, 41] despite being recommended and funded for many years. It is unclear whether uptake is so poor due to the vaccine not being offered to pregnant women, vaccine refusal or failure to record vaccination receipt. There is recent evidence to suggest that the introduction of the maternal pertussis vaccination program has been the impetus for driving up IIV uptake in pregnancy since its implementation in 2015 [42] although the data are lacking for NT Aboriginal women and sample sizes for Aboriginal women in other states were small or absent [40, 43].

Infants <7 m hospitalised with pertussis infections in this study were either unvaccinated with a pertussis-containing vaccine during their infancy or were too young to have completed at least two doses of a pertussis-containing vaccine in order to confer adequate protection from infection. A review of pertussis hospitalisations is warranted post implementation of the maternal vaccination program, with the expectation that there should be a decrease in notifications and hospitalisations in these youngest infants, given the 91–95% vaccine effectiveness that has been demonstrated elsewhere [23, 25] and provided adequate vaccine uptake in pregnancy is achieved. Pertussis vaccination in pregnancy could be particularly beneficial for Remote living Aboriginal mothers and infants from the Top End, as the disease burden was clearly highest in this population. To achieve this reduction in disease burden, the maternal vaccination program must be promoted and delivered in a culturally appropriate and respectful way [44, 45].

### Limitations

We acknowledge the potential biases that may arise from using routinely collected administrative data and the risk of their potential accuracy, as a limitation [46]. The impact of these limitations has been minimised through careful selection of the most reliable variables, restriction of data to avoid selection bias and the use of statistical techniques appropriate to observational data. The data integrating authority used for this study, SANT DataLink, reports >99% accuracy of its data linkage, based on reviews of all outputs using nationally agreed data quality checks [47]. Any potential mismatches in the linkage process are unlikely to have any impact on the representativeness of this whole of population observational study.

Notification data for pertussis and influenza infections were not included in the datasets, however, given hospitalisation is an indication of disease severity, the results from our large study can be used to infer the burden of severe disease in the NT by age, remoteness, region and Indigeneity. Further, while the small number of actual infant influenza and pertussis hospitalisations is a limitation, clearly the burden was heaviest for remote living Aboriginal infants from the Top End. The inability to capture the infants who would have moved to the region is a limitation of any birth cohort but is unlikely to have significantly impacted the data.

The NTIR did not record information for maternal vaccination status until 2010, given IIV in pregnancy was only recommended and not part of the funded scheduled immunisation program prior to this time. Unknown maternal vaccination status is, therefore, a limitation. There may be a small proportion of pregnant females aged 15–17 years from 2003 onwards (~<2%) who received a pertussis vaccination inadvertently as part of the school-aged dTpa booster vaccination program [3] however, we anticipate the actual number vaccinated to be low. There may also be a small proportion of Aboriginal females aged ⩾15 years from 2008 onwards (~11%) who received seasonal IIV in pregnancy as part of the annual recommended program for Aboriginal peoples, [48] although again we anticipate actual numbers of women who were vaccinated in pregnancy to be very low.

## Conclusion

We contribute whole of NT population baseline data for adverse birth outcomes and influenza and pertussis infections in early infancy in a pre-maternal vaccination era. Our study population comprises robust representativeness of Aboriginal and remote-living mother–infant pairs, demonstrating that Aboriginal women and infants potentially stand to benefit the most from maternal IIV and pertussis vaccination programs. In order to evaluate the impact of the maternal IIV and pertussis vaccination program of the NT, a subsequent review of the outcomes analysed in this paper, along with an assessment of vaccine uptake in pregnancy is required in the future.

## Supplementary material

For supplementary material accompanying this paper visit https://doi.org/10.1017/S0950268819001171. click here to view supplementary material
