# Supplementary material for: Baseline incidence of adverse birth outcomes and infant influenza and pertussis hospitalisations prior to the introduction of influenza and pertussis vaccination in pregnancy: a data linkage study of 78 382 mother–infant pairs, Northern Territory, Australia, 1994–2015
Source: Epidemiol Infect. 2019 Jul 4;147:e233. doi: 10.1017/S0950268819001171 (PMC6627012; doi:10.1017/S0950268819001171)
Supplement: Supplementary file 1 [file S0950268819001171sup.zip › S0950268819001171sup001.docx]

**Table 1:** Demographic characteristics in Northern Territory women by Indigenous status and remoteness,* Australia (1994-2014).

| **Characteristics** | | **Outer regional** | | **Remote** | | **Very remote** |
| --- | --- | --- | --- | --- | --- | --- |
| **Aboriginal (n=27 420)** | | **n=7162 (26%)** | | **n=9888 (36%)** | | **n=10 370 (38%)** |
| Median maternal age at infant birth (range)  Mother <20 years of age | | 24 (12-51)  1387 (19%) | | 23 (12-46)  2666 (27%) | | 22 (12-44)  3121 (30%) |
| 1^st^ trimester attendance for antenatal care | | 2914 (45%) | | 3607 (39%) | | 2963 (31%) |
| Smoking in pregnancy (36 weeks gestation) | | 1477 (43%) | | 2298 (46%) | | 1954 (38%) |
| Consuming alcohol (36 weeks gestation) | | 484 (10%) | | 527 (8%) | | 539 (8%) |
| Maternal anaemia | | 410 (6%) | | 1010 (10%) | | 1047 (10%) |
| Cardiac disease | | 184 (3%) | | 571 (6%) | | 530 (5%) |
| Hypertension | | 80 (1%) | | 135 (1%) | | 180 (2%) |
| Pre-eclampsia | | 265 (4%) | | 435 (4%) | | 687 (7%) |
| Renal disease | | 75 (1%) | | 242 (2%) | | 244 (2%) |
| Gestational diabetes | | 236 (3%) | | 362 (4%) | | 390 (4%) |
| Urinary tract infection | | 250 (3%) | | 574 (6%) | | 669 (6%) |
| Sexually transmissible infection | | 71 (2%) | | 143 (2%) | | 234 (3%) |
| **Non-Aboriginal (N= 49 108)** | **n=41 214 (84%)** | | **n=5143 (10%)** | | **n=2751 (6%)** | |
| Mean maternal age at infant birth (range)  Mother <20 years of age | 29.2 (13-50)  1779 (4%) | | 28.6 (13-47)  324 (6%) | | 29.2 (14-45)  118 (4%) | |
| 1^st^ trimester attendance for antenatal care | 23 705 (63%) | | 2780 (60%) | | 1547 (59%) | |
| Smoking in pregnancy (36 weeks gestation) | 3162 (14%) | | 668 (22%) | | 260 (16%) | |
| Consuming alcohol (36 weeks gestation) | 867 (3%) | | 246 (6%) | | 81 (4%) | |
| Maternal anaemia | 601 (1%) | | 109 (2%) | | 60 (2%) | |
| Cardiac disease | 241 (0.58%) | | 46 (0.89%) | | 37 (1.34%) | |
| Hypertension | 251 (0.61%) | | 47 (0.91%) | | 29 (1%) | |
| Pre-eclampsia | 1530 (4%) | | 181 (4%) | | 118 (4%) | |
| Renal disease | 159 (0.39%) | | 25 (0.49%) | | 14 (0.51%) | |
| Gestational diabetes | 828 (2%) | | 100 (2%) | | 51 (2%) | |
| Urinary tract infection | 454 (1%) | | 120 (2%) | | 70 (3%) | |
| Sexually transmissible infection | 69 (<1%) | | 16 (<1%) | | 16 (<1%) | |

**Note:** denominators differ due to missing data.

* Australian Bureau of statistics designated SLA regions: incorporates Darwin city and surrounding suburbs and Alice Springs urban

† In the Northern Territory of Australia
