# Supplementary material for: Baseline incidence of adverse birth outcomes and infant influenza and pertussis hospitalisations prior to the introduction of influenza and pertussis vaccination in pregnancy: a data linkage study of 78 382 mother–infant pairs, Northern Territory, Australia, 1994–2015
Source: Epidemiol Infect. 2019 Jul 4;147:e233. doi: 10.1017/S0950268819001171 (PMC6627012; doi:10.1017/S0950268819001171)
Supplement: Supplementary file 1 [file S0950268819001171sup.zip › S0950268819001171sup002.docx]

**Supplementary table 2:** Maternal and infant birth outcomes in Northern Territory women, by Indigenous status and remoteness, Australia (1994-2014).

| **Characteristics N (%)** | **Outer regional** | **Remote** | **Very Remote** |
| --- | --- | --- | --- |
| **Aboriginal (N= 27 420)** | **n=7162 (26%)** | **n=9888 (36%)** | **n=10 370 (38%)** |
| Median gestation in weeks (range) | 39 (20-43) | 39 (20-43) | 39 (20-43) |
| Mean birthweight in grams (range) | 3211 (400-5834) | 3058 (400-5490) | 3064 (400-5660) |
| Preterm birth (<37 completed weeks) | 919 (13%) | 1594 (16%) | 1641 (16%) |
| Low birthweight at term* | 241 (4%) | 521 (6%) | 487 (6%) |
| Small for gestational age^†^ | 1098 (15%) | 1987 (20%) | 2034 (20%) |
| Stillbirths | 98 (1.37%) | 112 (1.13%) | 166 (1.60%) |
| **Non-Aboriginal (N=49 108)** | **n=41,214 (84%)** | **n=5143 (10%)** | **n=2751 (6%)** |
| Median gestation in weeks (range) | 39 (20-43) | 40 (20-42) | 40 (21-42) |
| Mean birthweight in grams (range) | 3354 (400-5980) | 3344 (420-5330) | 3420 (400-5040) |
| Preterm birth (<37 completed weeks) | 3148 (8%) | 396 (8%) | 196 (7%) |
| Low birthweight at term* | 711 (2%) | 96 (2%) | 30 (1%) |
| Small for gestational age^†^ | 4597 (11%) | 598 (12%) | 252 (9%) |
| Stillbirths | 268 (0.65%) | 31 (0.60%) | 15 (0.55%) |

**Note:** denominators differ due to missing data.
